# Description and evolution of a *nimB* gene-coding, composite mobilizable transposon, MTn*Bf8*, of *Bacteroides fragilis*

**DOI:** 10.3389/fmicb.2026.1923328

**Published:** 2026-09-09

**Authors:** József Sóki

**Affiliations:** Institute of Medical Microbiology, Albert Szent-Györgyi Medical School and Health Centre, University of Szeged, Szeged, Hungary

**Keywords:** *Bacteroides fragilis*, insertion sequence elements, integrases, metronidazole resistance, mobilizable transposon, *nimB*

## Abstract

**Background:**

In *Bacteroides* strains, metronidazole resistance is encoded mainly by the so-called *nim* genes, which are usually carried on plasmids; however, chromosomal presence is also known (e.g., *nimB*). Usually, the *nim* genes are activated by insertion sequence (IS) elements, conferring resistance. Molecular epidemiological observations predict that this configuration most likely arose before the formation of the best-known carrier mobile genetic elements.

**Objective:**

To determine the *nimB* genetic element and study its evolution.

**Methods:**

To fulfill the goals, the whole-genome sequence of *B. fragilis* BF8, inverse PCRs, and bioinformatic analyses were used.

**Results:**

A novel mobilizable transposon, MTn*Bf8*, is described, with an integrated part, Tn*NimB1*, that carries the chromosomal *nimB* gene. Cassettes similar to Tn*NimB1* were found in *Bacteroides*-related species in fecal metagenomes, but these species did not harbor IS*1168*. In inverse PCR, the excised intermediates of MTn*Bf8* and Tn*NimB1* were detected, thereby confirming their mobility. In alignments of nucleotide and protein sequences, the existence of the Tn*NimB*s and at least three groups of integrative elements of *Bacteroides* could be confirmed, and a scheme for the emergence of different *nimB-*carrying elements was drawn.

**Conclusion:**

The data presented here could explain the above-mentioned emergence of MTn*Bf8*/Tn*NimB1* and the IS-*nimB* configurations and their expected spread.

## Introduction

*Bacteroides* species are among the most important and abundant anaerobic bacteria associated with the human body, as they are major constituents of the gastrointestinal microbiota and are also endogenous opportunistic pathogens (Wexler, 2007). They are also the most antibiotic-resistant anaerobes, including the anti-anaerobic 5-nitroimidazoles (e.g., metronidazole), which are caused by the so-called *nim* genes in most cases. The *nim* genes have 13 distinct representatives (*nimA-L*) and can be carried on both plasmids (*nimA*, *C-E*) and chromosomes (*nimB*, *J*) (Alauzet et al., 2019b). In resistant cases, they are upregulated by insertion sequence (IS) elements, and these associations usually involve a specific *nim* gene-IS element pair (e.g., *nimA*-IS*1168*) (Sóki, 2013; Sóki et al., 2006). Additionally, the distance between the *nim* genes and their activating IS elements is almost always constant (for *nimA*-IS*1168*, it is 19 nt) (Sóki et al., 2006), suggesting that these configurations most likely arise before reaching the carrying replicons, but information on how they emerged is lacking.

The association of *nimB* and IS*1168*, and their chromosomal location, was described early, when the *nimB* gene was discovered (Haggoud et al., 1994), but the specific genetic element carrying them, unlike some plasmids, has not been identified. Additionally, a cluster of multidrug-resistant *B. fragilis* strains has been identified that carries, in addition to the *nimB*-IS*1168* pair, the chromosomal carbapenem resistance gene *cfiA* and the IS*4351* element (Sóki et al., 2016). Genome sequences were determined for some representatives of this cluster of *B. fragilis* strains (Patrick et al., 2010; Sóki et al., 2016).

The *Bacteroides* also harbor a set of integrating mobile genetic elements, such as conjugative and mobilizable transposons (CTns and MTns), regular transposons, and insertion sequence elements (Smith et al., 1998). Regarding the conjugative transposons, CTnDOT, CTnGerm, CTnBST, CTn12256, and CTn341 have been well characterized (Husain et al., 2014), despite the fact that a vast amount of nucleotide sequence data is now available in the databases for tetracycline resistance-coding CTns. Some short (<15 kb) MTns are also characteristic of *Bacteroides* strains and genomes. These include the so-called non-replicated *Bacteroides* units (NBU1 and NBU2) discovered in the early 1990s (Stevens et al., 1992), the cefoxitin resistance gene carrier MTn*4555* (Tribble et al., 1997), and the less well characterized “ermF region” of CTnDOT (Whittle et al., 2001), MTn5520 (Vedantam et al., 1999), and clV25 (Bass and Hecht, 2002). We also have substantial data on nim-gene-carrying plasmids (e.g., pIP417, pIP419, pIP431, pBF388c) (Reysset, 1996; Sóki et al., 2006).

Such integrative elements typically undergo site-specific integration and excision to move between different cellular locations. The integrase proteins that carry out these processes of these mobile genetic elements (MGEs) belong to the so-called TSSRs (tyrosine site-specific recombinases) (Craig, 2015; Jayaram et al., 2015). These were classified into well-defined groups based on their amino acid sequences and tertiary structures in recent studies. Thus, a scheme could be established for the pairs of the integrase protein types and the integrative chromosomal elements that carry them (Smyshlyaev et al., 2021).

In this work, the aim was to define the genetic element of the *nimB* gene and to determine the origin and emergence of the *nimB*-IS*1168* pairs during evolution, primarily by identifying homologous elements and using TSSR phylogeny. The results support the peculiarity and establishment of the *nimB*-carrying MGEs and other *nim*-IS combinations, too.

## Materials and methods

### Bacterial strains and cultivation

The *nimB*-carrying *B. fragilis* strains BF8 and KSB-R (Sóki et al., 2016) were used and stored in BHI broth supplemented with 20% glycerol at −80 °C. Cultivations were done on Columbia Anaerobic agar plates (Columbia agar base supplemented with 5% sheep blood, 2.5% laked sheep blood, 0,3 g/l cysteine and 1 mg/l vitamin K1) or in supplemented BHI broth (BHIS, BHI broth supplemented with 0.5% yeast extract, 5 mg/l haemin and 1 mg/l vitamin K1) under anaerobic conditions (85% N_2_, 10% H_2_ and 5% CO_2_, in a Whitley A45 Anaerobic Workstation (Bently, UK) at 37 °C (for 48 h for agars and 24 h for BHIS broth).

### Bioinformatic applications and localization of the proposed “*nimB* element”s’ sequences of *B. fragilis* BF8

For handling, visualizing, and analyzing nucleotide sequences, the features of the Artemis (Welcome Trust),^1^ Islandviewer 4^2^ (Bertelli et al., 2017), Lasergene 17 (DNAStar, Madison, WI, USA), and Snapgene^3^ software were used, and BLAST searches^4^ were also applied.

The larger *nimB*-harboring genetic element was determeined in two ways: (i) examination of the annotated chromosomal neighborhood of the *nimB* genes in Artemis and (ii) genomic island detection by Islandviewer 4. The expected left and right ends for the element was aligned by ClustalW (Lasergene 17), which was experimentally proven by excision PCR methods (see next section).

For determination of the smaller *nimB* element MAUVE alignments were used (ClustalW, Lasergene 17).

### PCR methods applied to detect the excised forms of the “*nimB* elements”

To carry out inverse PCRs for the detection of excised and circularized forms, the strains were cultivated overnight in 5 ml of BHIS broth with tetracycline (1 μg/ml), metronidazole (1 μg/ml), moxifloxacin (0.4 μg/ml), and mitomycin C (0.1 μg/ml) induction or without it. After this, the cultures were refreshed in 5 ml of BHIS broth at 1/40 dilution and allowed to grow anaerobically for further 4 h. Then to prserve the integrity of the excision forms the following cell preparation was applied; 10 μl of culture was centrifuged at 12000 rpm for 1 min at room temperature, the broth was decanted, and the cell pellet was resuspended in 10 μl of sterile UP water (Franco, 2004). 5 μls of this were used in 25 ul PCR mixtures in end-point PCR reactions (12.5 μl 2× DreamTaq mastermix, Fermentas, with 0.7 μM primer concentrations). The sequences of the PCR primers and cycling conditions are given in Supplementary Table 1. The products were analyzed by 1.2% agarose gel electrophoresis (0.5× TBE buffer with 0.5 μg/ml ethidium bromide staining) and, if needed, purified by PCR cleanup and sequenced by fluorescent capillary sequencing (Sóki et al., 2013, 2016).

Real-Time PCR was used to determine the excision rate of the MTn*Bf8*. Composition of the PCR reactions was as follows: 5 μl QuantiNova RT-PCR master mix (Qiagen), 0.7 μM primers, and 2 μl uninduced cell suspension prepared as described above. Primer sequences and cycling conditions are given in Supplementary Table 1.

## Results

### Description of MTnBf8

**FIGURE 1:**
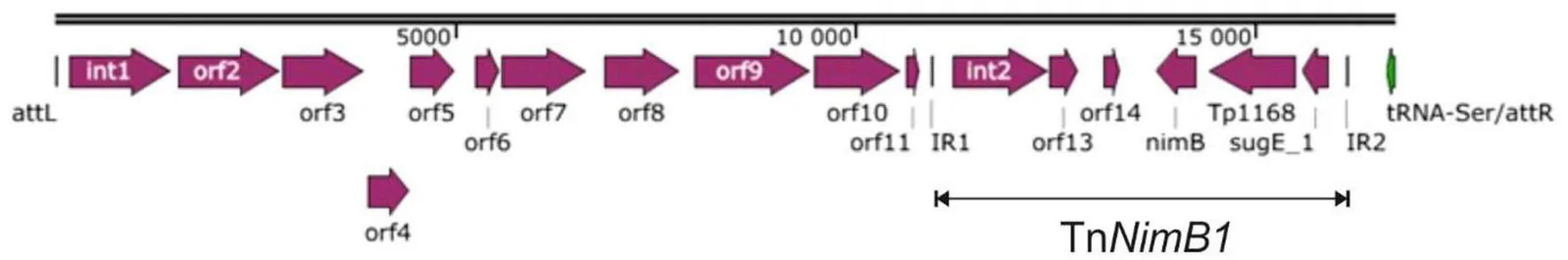
The genetic map of MTn*Bf8* with Tn*NimB1.*

It was not well known which genetic elements the chromosomal *nim* genes, especially *nimB*, a *nim* gene discovered among the first, reside on. However, the whole genome nucleotide sequences have been determined for some of them, e.g., *B. fragilis* BF8 and O:21 (Patrick et al., 2010; Sóki et al., 2016). In determination of the MGE for *nimB* near the single chromosomal *nimB* locus, two ORFs annotated as XerCD proteins and a Ser-tRNA gene were localized, resembling the case of some MTns (NBU1 and NBU2) in *Bacteroides*. Then an alignment of the possible ends was made by ClustalW, which revealed two strongly homologous direct repeats (Supplementary Figure 1). By further examination of the ORFs between these two direct repeats, the structure of an MTn has been revealed by carrying integrase (*int1* and *int2*), and mobilization (*mob*) functions with other genes, including the *nimB*-IS*1168* pair too (the genetic map and the list of ORFs are shown in Figure 1 and Table 1). Islandviewer 4 also detected this region (data not shown). It was interesting that the *nimB* gene was located on a segment bordered by inverted repeats, which also included the second integrase gene (*int2*). To prove the MTn nature and the capability for excision, an outward-oriented primer pair (Supplementary Figure 2 and Supplementary Table 1) was designed, and inverse PCRs were carried out with tetracycline, metronidazole, moxifloxacin, and mitomycin C induction and without it. Using these primers, PCR products were obtained in equal amounts under all of the above conditions (Supplementary Figure 3). Sequencing these PCR products confirmed that the ends of the proposed MTn are joined during an excision process (Supplementary Figure 1) and therefore this confirmed element was termed MTn*Bf8* (after type strain *B. fragilis* BF8). Since the excision of MTn*Bf8* was constitutive (Supplementary Figure 3), the approximate rate of this was determined in an RT-qPCR experiment (Supplementary Figure 4). This gave an estimate that ca. 6% of cells have MTn*Bf8* in the excised form.

**TABLE 1:** The main features of MTn*Bf8*.

Feature no.	Start in BF8 genome	Start in MTn*Bf8*	Length nt	Length aa	Direction	Feature name	Feature information	Feature additional information
1	598671	1	25			*attL*		
2	598845	174	1283	428		*int1*		
3	600197	1526	1745	582		*orf2*	DUF1016	Restriction endonulease-like RecB
4	601500	2829	1040	347		*orf3*	hyp	
5	602569	3898	542	181		*orf4*	hyp	
6	603101	4430	557	186		*orf5*	hyp	
7	603925	5254	308	103		*orf6*	DUF3853	
8	604248	5577	1076	359		*orf7*	P-loop NTP-ase/mobilization	AAA domain; *recA* -TrwB
9	605530	6859	956	319		*orf8*	Topoisomerase primase	
10	606658	7987	1457	486		*orf9*	DUF/mobilization-segregation	mobN1, mobilization protein
11	608156	9485	1091	364		*orf10*	DKNNYY	Fusobacterial 35 repeats (membrane)
12	609298	10453	347	116		*orf11*	DUF4304	
13	609618	10947	23			IR1		
14	609879	11208	1208	403		*int2*		
15	611099	12428	371	124		*orf13*	*rhuM*	Salmonella SPI-3
16	611781	13110	203	68		*orf14*	hyp	
17	612426	13755	494	165	c^a^	*nimB*		
18	613098	14427	1067	356	c	*Tp1186*		
19	614808	16137	23		c	IR2		
20	614257	15586	320	107	c	*sugE*		Cation efflux EamA EmrE SMR efflux
21	615300	16629	25			*attR*		
22	615302	16631	85	–	–	tRNA-Ser	–	–

*^a^*Coded on the complementary strand.

In addition, a region was disclosed that harbored *int2* and *nimB* bordered by an inverted repeat pair (IR1 and IR2, Figure 1, Table 1, and Supplementary Figure 5).

### Emergence of some specific regions harboring the *nimB* gene on MTnBf8 and on other DNA segments

To elucidate the function of *int2*, the possibility that it is responsible for excising only the *nimB*-carrying part (Tn*NimB1*) of MTn*Bf8* was investigated. This way, outward-oriented primers located at the ends of Tn*NimB1* and more specifically in the *nimB* gene (Supplementary Figure 2 and Supplementary Table 1) were used in end-point PCR reactions. In case of the first primer pairs, a constitutive, but less abundant than for the whole MTn*Bf8* product, was obtained again - excision of Tn*NimB1* (data not shown). However, no products were yielded with the second primer pair - excision of the *nimB* gene alone (data not shown). Sequencing the PCR product from the excised Tn*NimB1* confirmed that the inverted repeats indeed define its ends and that the insertion involves a coupling sequence (Supplementary Figure 5).

Next, the emergence of the *nimB*-IS*1168* composition and the origin of the Tn*NimB1* part were also approached. As we hypothesized, the *nimB*-IS*1168* constitution arose before it was assembled into a mobile genetic element and subsequently spread. To examine this possibility, the Tn*NimB1* part was megablasted against all genome sequences (both isolated and metagenomic Bacteria records) from GenBank. Interestingly, three hits were obtained all from Bacteriodota; one from a *Mediterranea* isolate genome (*Mediterranea* sp. An20), one from an *Alistipes* sp. genome (*Alistipes* sp. An54) (Medvecky et al., 2018), and the third from an *Alistipes communis* genome (*Alistipes communis* MAG168) (Tang et al., 2026). All these MAGs (metagenome-assembled genomes) were from animal fecal microbiotas and carried segments with ca. 100% identity to Tn*NimB1*, except that the IS*1168* was missing and an additional variation at the 3′ end. The common ORFs were as follows: an integrase (*int2*), two ORFs annotated as *rhuM* and a hypothetical protein gene, *nimB*, and a cation efflux pump gene (*sugE*). The alignment of the three MGEs with MTn*Bf8* is shown in Supplementary Figure 5A. The alignment of these four sequences was consistent with the excised form sequence; it ended exactly at the proposed inverted repeats (Supplementary Figure 5B).

In addition to these, some short *nimB* gene-carrying segments were also found in the databases – an exceptional *nimB* small plasmid (Boiten et al., 2023) and another, possibly chromosomal, segment that had the *nimB*-IS*1168* pair on a shorter span (GenBank acc. no. NZ_JBHBMX010000031) (Supplementary Figure 6A). While this segment ended on the right end just at the IS terminal inverted repeat of IS*1168*, on the left end, some similar inverted repeat sequences could be found, which may define some ends for this short element (Supplementary Figure 6B).

**FIGURE 2:**
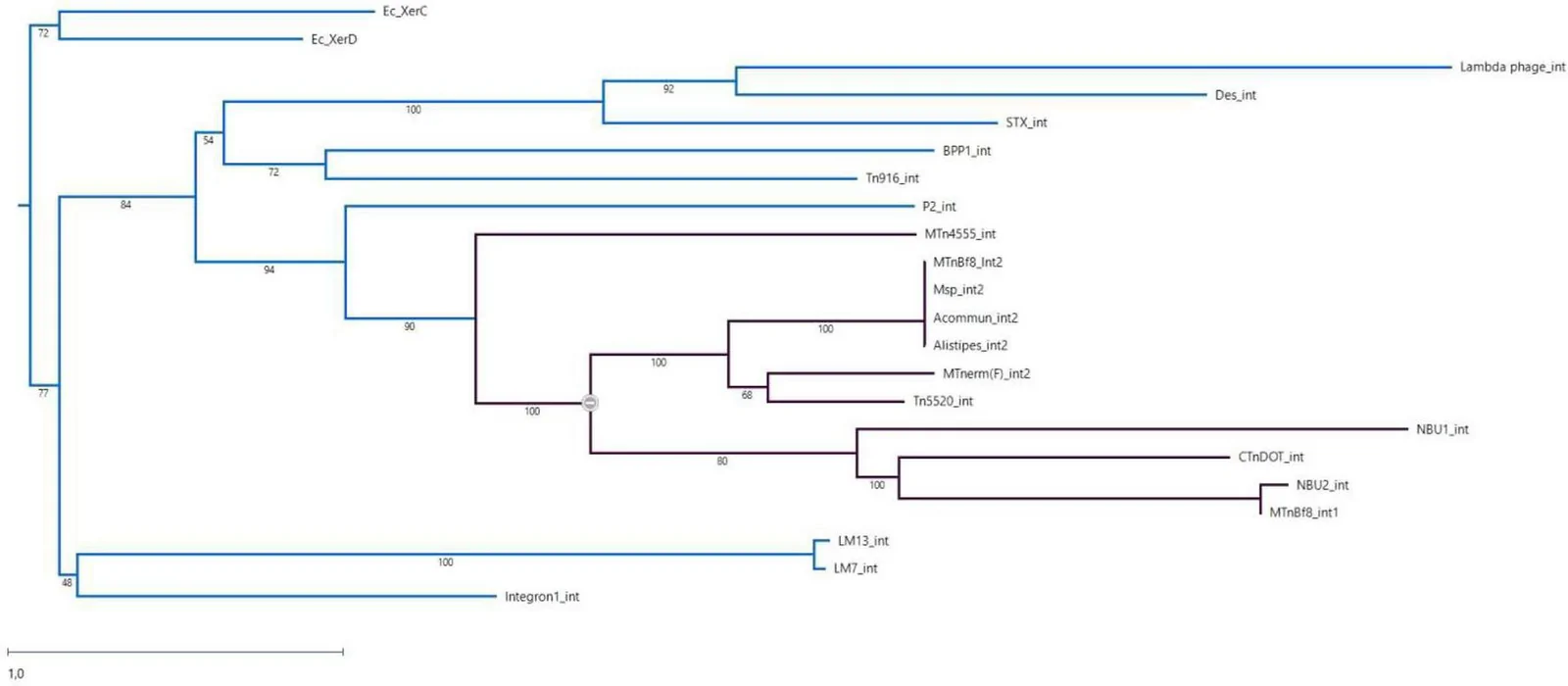
Phylogenetic tree of some TSSR group representatives with those of *Bacteroides.* Alignment of the protein sequences for tree construction was done by the Clustal W algorithm (lasergene 17, default parameters with 1000 bootstrapping), the source files from top to down are: NP_418256.1, NP_417370.1, NP_040609, EMB03627, WP_174405645, NP_958719, CWW49344, AAO64735, GCA_001695355.1, BSF34_RS08435, WP_065739606, B5G09_Rs13525, AJ311171, AAC80279, AAB06447, AJ311171, AF251288_3, GCA_001695355.1, KU366690, KU366689. The branches of the *Bacteroides* sequences included are in black color, the numbers at the branches give the bootstrap values and the bar for the distances is also shown below.

According to the original hypothesis and to further investigate the origin of Tn*NimB1*, a phylogenetic analyses of the integrases (Int2s) was conducted, assuming that Int2s are genuine representatives of Tn*NimB*s and that Tn*NimB*s are the early *nimB* vehicles and maintain the *nimB* gene pool in microbiotas containing Bacteroidota. But first, the Int2 protein-blasting classification assigned the Int2s to the INTN-1-C/BRE-C group of tyrosine recombinases. Based on the classification scheme of Smyshlyaev et al. (2021) for different MGE Int proteins, different groups of TSSR sequences and the well-characterized *Bacteroides* integrases were included in a phylogenetic analysis (Figure 2). This gave an agreement with the above MGE classification scheme and also revealed that all *Bacteroides* TSSRs belong to a distinct subgroup of INTN-1-C. Moreover, this analysis showed that the *Bacteroides* Int proteins formed three close but distinct branches, including MTn*4555*, and two other groups defined by Int1 and Int2. Thus, according to our view, there are at least three types of integrative (mobilizable/conjugative) transposon groups in *Bacteroides* (Figure 2) if we assume that, during their evolution, they did not change their TSSRs.

## Discussion

In this study, a segment of chromosomal DNA was predicted and subsequently confirmed by inverse PCR to be a mobilizable transposon. Thus, the transposon was ca. 16.3 kb long, and another feature, that it is composite, has also been demonstrated. Two tyrosine integrase genes, *int1* and *int2*, were detected, with *int2* belonging to a module called Tn*NimB1*. MTn*Bf8* showed similarities (in approximate size, *int1* and *orf9* being highly homologous to the NBU *int* and *mob* functions, respectively) and dissimilarities (no *exc*, two *mob*s, and an embedded other element) to the known representatives of *Bacteroides* MTns. MTn*Bf8* was also unique in some respects compared to the known *Bacteroides* MTns. It did not harbor an *exc* function but harbored two possible *mob* genes (*orf7* and *orf9*) and other functions that could be involved in virulence or whose functions were unknown (Table 1).

The inverse PCR experiments confirmed that the observed direct and inverted repeats define these elements and provided information on the expected excision-integration mechanisms. The whole MTn*Bf8* uses *att*-like sequences, while the integrated Tn*NimB*s apply inverted repeats and coupling sequences between them. These points could be analysed further with the mobility proposal of the small *nimB*-IS*1186* “element.”

Despite positive results across the MTn*Bf8* inverse PCR experiments, horizontal gene transfer could not be achieved in earlier experiments for eight of the *nimB*-IS*1168*-carrying *B. fragilis* cluster strains. At present, we explain that these strains may not harbor a complete and functional set of *tra* genes (as supported by preliminary observations in the genome sequences).

This study defined a complete chromosomal nim MTn element for the first time. Earlier work on the nimJ gene determined its localization on a CTn (CTnHyb). However, CTnHyb harbored additional antibiotic resistance traits, mostly of Gram-positive origin, that were also transferred via conjugation, a process known as the “Ellis Island effect” (Husain et al., 2014).

In addition, as the specialty of MTn*Bf8*, Tn*NimB1* appears to be the possible ancestral reservoire of the *nimB* gene – it was found in other locations in some Bacteroidota genomes (*Alistipes* and *Mediterranea* strains) with small variations. It is also known that other *Bacteroides* relatives, including all *Prevotella baroniae* strains, harbor the *nimI* gene (Alauzet et al., 2010). Therefore, we might expect that other anaerobic Bacteroidota strains can also carry additional *nim* genes. *Nim*s are relatives of the pyridoxamine-phosphate oxidoreductase (PPOR) proteins (Alauzet et al., 2019a), so we can also hypothesize that their evolution from the PPOR ancestor occurred in e.g., Bacteriodota species, giving rise to antibiotic resistance genes. However, to confer resistance, *nim*s require IS element insertions that maintain a constant expression level under variable conditions (Mahmood et al., 2023). This is not an exception for *Bacteroides;* the expression of many *Bacteroides* antibiotic resistance genes [e.g., *cfiA*, *cfxA*, and *erm*(F)] is upregulated by IS elements (Sóki, 2013). But in the case of the carbapenemase coding *cfiA* gene, there are silent forms in a subset of *B. fragilis* strains, which are turned to cause resistance by IS insertions in about 1%–10% of the total *cfiA*-positive cases (Sóki, 2013). This is markedly different from the situations of *nim*s. The majority of the analyzed *nim*-mediated metronidazole-resistant *Bacteroides* strains originally carry a more or less IS element specific to the *nim* gene (Sóki et al., 2006). It was already mentioned that in these configurations, the distance of the *nim*-IS pair is conserved. This led to the hypothesis that IS insertion occurs before integration into replicons and subsequent spread. In this study, some traces proving this were also found – the *nimB*-IS*1168* segments on the pBspL04_3 plasmid (Boiten et al., 2023) and on a related element. Here, MTn*Bf8* could have been the intermediate between these locations and the “original” carrier elements Tn*NimB*s. Tn*NimB1* might function like an integron due to the presence of *int2* and several antibiotic resistance genes. However, excision experiments could not support it; *nimB* was not on an integron cassette-like segment. Still, the study of Smyshlyaev et al. (2021) found that Bacteriodota harbor integron integrase-like genes in a significant proportion. Phylogenetic tree construction confirmed that the known TSSR genes on the well-characterized MGEs of *Bacteroides* form three branches. Which were as follows (i) MTn*4555*, (ii) CTnDOT/NBU, and (iii) *int2* for short or exceptional elements (TnNimBs and MTn*ermF*). In the phylogenetic tree shown here, the integrases classified by Smyshlyaev et al. (2021) separated well, supporting the conclusion that an appropriate tree was drawn. These latter proteins, including the INTN-1-C types (mentioned above), can be divided into three main segments: AB – arm-binding domain, CB – core-binding domain, and CAT – catalytic domain, of which the first (AB) is missing from most TSSRs, including the integron integrase. This further supports the conclusion that, although integron integrase-like genes are found in Bacteriodota (Smyshlyaev et al., 2021), the *int2* gene described here does not function like them but rather participates in the mobility of the Tn*NimB* elements.

**FIGURE 3:**
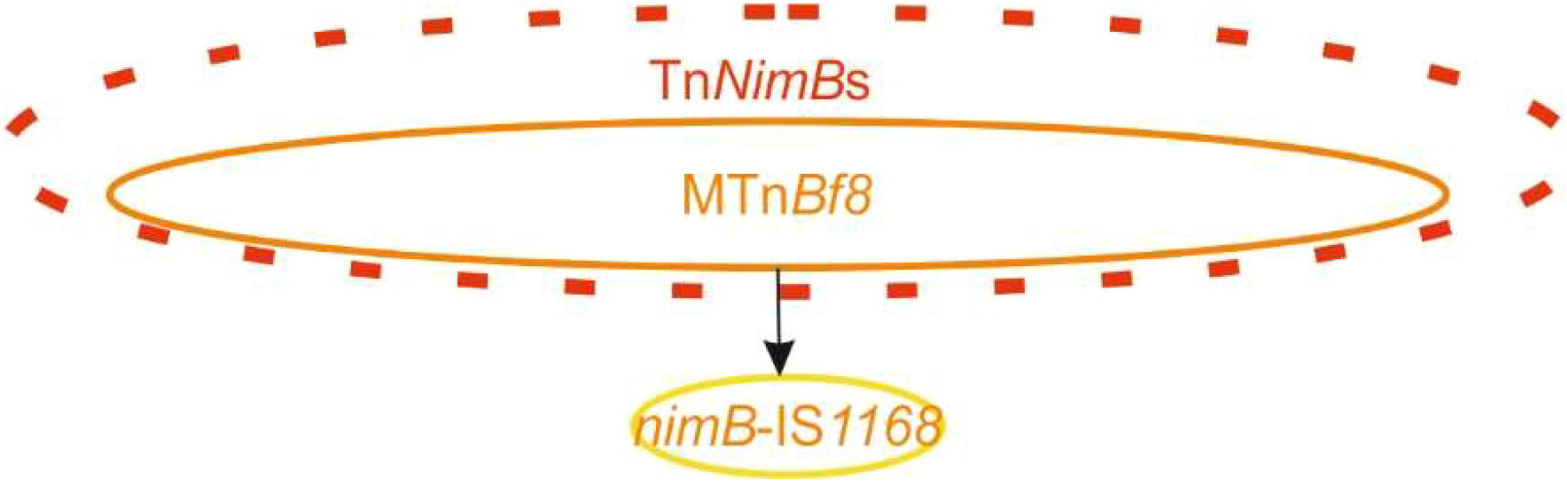
A scheme for the development of the “*nimB* elements”. A microbiota pool harbors the possible ancestral reservoire of *nimB* elements (its size is unknown and they may be Tn*NimB*s; marked with dashed red line); the MTn*Bf8* element is spread among *Bacteroides* strains by its resistance causing property due to the IS*1168* insertion (orange ellipse), the *nimB*-IS*1168* configuration skipped to some other MGEs (yellow ellipse).

From the data presented here, the following scheme could be proposed for the emergence and evolution of the *nimB* MGEs: 1. various elements of Tn*NimB*s harbor the *nimB* gene among Bacteriodota, 2. of which one, MTn*Bf8* picked up IS*1168* and has spread widely, and 3. a smaller segment is transferred to other replicons (Figure 3). However, it is only one possible scenario which has the most probability at present time. But this might have happened to other *nim* genes as well, since they are carried on small segments of other frequent replicons/plasmids; still, the distances between the *nim* genes and the IS elements are conserved.

While the spread of the *nimB* gene can be described as outlined above, we have just a few representatives from MAGs. May-be, this rarity will be ameliorated with time as the of genomic sequence databases become more abundant, e.g., they improve by improving rarefaction. This warns us to follow the pop-up of other similar sequences from the ubiquitous microbiotas containing Bacteroidota strains and also to catch somehow the mobility of the *nimB*-IS*1168* pair.

### Summary

A novel mobilizable transposon, MTn*Bf8*, was described here, and, by detecting excision intermediates and aligning homologous sub-sequences, it was also characterized in some detail. The data also suggest a way in which the frequent *nim* gene-carrying replicons might arise, and there could be at least three different subtypes of INTN-1 MGEs among Bacteroidetes.

## Data Availability

The datasets presented in this study can be found in online repositories. The names of the repository/repositories and accession number(s) can be found in the article/Supplementary material.

## Footnotes

1
https://sanger-pathogens.github.io/Artemis/

2
https://www.pathogenomics.sfu.ca/islandviewer/

3
https://www.snapgene.com/

4
https://blast.ncbi.nlm.nih.gov/Blast.cgi
