# Supplementary material for: Description and evolution of a nimB gene-coding, composite mobilizable transposon, MTnBf8, of Bacteroides fragilis
Source: Front Microbiol. 2026 Sep 9;17:1923328. doi: 10.3389/fmicb.2026.1923328 (PMC13597742; doi:10.3389/fmicb.2026.1923328)
Supplement: Supplementary file 1 [file Table_1.docx]

**Supplementary material**

**Fig. S1.** Alingnment of the expected ends of MTn*Bf8*

```
right     --------------------------gattagcccataaccctttaaataagaaaacacc
left      ccggtcaaaataatataatttccatcaatacggcaatgtctgatta-----taaaatggt
                                     **  * * **  *   ***      ****
right     cgactttacattcttgggtgtaaaatcgggtgtttatttt--------------------
left      agcattatcatacctttttattcactcaggtgttctggaaataaaaagtataaatagtaa
           *  **  *** * *   * *  * ** ******
```

right	------------------------------ataaaacattgattttcaatgtttatt*gcg*

left	aaagcctacaagttgtgaacttgtaggctttttatccacttaccatccgataagtttgcg

* * ** * *	**	*****

```
right     gagagacagggattcgaaccccgggtacctcgcagtacaacggttttcaagaccgccgca
left      gagagacaggctctcgaacctgcgctatctcaaaatatcacacaatcgcaaatatc----
          **********   *******   * ** ***  * **  **    *   * *   *
right     atcgaccactctgccacctctccaaaactacttttc------------------------
left      ---tattagtaaccaacttattacaatcataattaaatctaaatattatcgaatatcgct
              *  * *   * ** * *   ** *    **
right     ------------------------------------------------------------
left      cgctatttcaatttttgggtgtatatttgggtgcagaatttcaaatacacccagtcatga

right     ----------------------------------------------------
left      acatcaaacgaaacatcatttttgcacctgaaagccgcaaaaagaacggcgt
```

The left and right bordering sequences of MTn*Bf8* carrying the *attR* and *attL* sequnces (boxed) are shown. The sequence of the excised closed circular form is shown in red, and the tRNA-Ser sequence is in italic in *attR*.

**Fig. S2.** The positions of inverse primers to detect excisions of MTn*Bf8*, Tn*Nim1 and nimB*

**Table S1.** Sequences of PCR primers and the parameters of the PCR cycles

| Name | Sequence 5'→3' | PCR cycles |
| --- | --- | --- |
| nimBeR1 | GTCTTTTTGCGGCTTTC | 94 °C 150 s; 94 °C 15 s, 59 °C 30 s, 72 °C 2 min 35x; 72 °C 5 min (end-point PCR) |
| nimBeF1 | GTCAGGTCTAAACAAAAAT |  |
| nimBeR2 | CAAGACAGAAAGCAAAAG |  |
| nimBeF22 | GACTGTCTTGAAAGTGCTCCTC |  |
| I_nimB_1 | GTATGCCGTTCCCATCAGTT |  |
| I_nimB_2 | TCTGCCAATAAACCCAAAGC |  |
| nimBeF1 | see above | 95 °C 10 min; 95 °C 1 min, 51 °C 1 min, 72 °C 2 min 35x (RT-PCR) |
| attR-Tn8 | AAAATGCCGAAACGTTTGCA |  |

**Fig. S3.** Picture from an excision PCR experiment for the whole MTn*Bf8*

1	2	3	4	5	6	7	8	M

←from circularized *MTnBf8*

Lanes: 1-4 – B. fragilis BF8, 5-8 – B. fragilis KSB-R, 1, 5 – no induction, 2, 6 – moxifloxacin induction, 3,7 – tetracycline induction, 4, 8 – mitomycin C induction. M: 100 bp ladder plus (100, 200, 300, 400, **500**, 600, 700, 800, 900, **1000**, 1200, 1500, 2000 and 3000 bp)

**Fig. S4.** Estimation of the amount of the constitutively excised form of MTn*Bf8*

↑	↑

C_T_	*attR*	cir

A *ca.* 4-step difference in C_T_ values gives 6.25 % (1/16) constitutively excised form ratio

**Fig. S5.** Alignments and end sequences of the Tn*NimB*-carrying genetic elements

A

↑

Tn*NimB*s

B

WWTGGAAAAGAATAGGATAAAGAGCAAAAATAAGCGAATTTGAGCA.. TTGTTTATTTTTAATATATTCCTATTTACATATCGGATTTT

(A) MAUVE alignment done with Lasergene 17 of the TnNim element carrying sequences found in Bacteroidetes genomic sequences. (Details of the last three: Mediterranea sp. An20 [NZ_NFJV01000012], Alistipes sp. An54 [NZ_NFIA01000024] and Alistipes communis MAG168 [NZ_DBMEMY010000002]). Location of Tn*NimB*s are marked with an arrow. (B) The end sequences of the Tn*NimB*s from the alignmnent and from the ‘inverse’ PCR sequencing. Coupling sequence is marked in purple and inverted repeats, found by a Clustal W algorithm (Lasergene 17, default parameters) are marked with green colour.

**Fig. S6.** Alignment and end sequences of the small *nimB* segments

A

↑

*nimB*-IS*1168*

B

GGGGACTTCTATAAATTAAATCCCTGATTATAAGTTGCATA.. CAGGGGAACCGAACGTCCGTTATTTGATTTCGAACTCCTCCT

consensus: GGAnTTC..AAATnAAAT

(A) MAUVE alignment (lasergene 17) of some shorter forms of *nimB* harbouring genetic segments (source files: NZ_CP119605, NZ_JBHBMX010000031). The homologous regions are marked with an arrow. (B) Alignments of the ends of the short *nimB* segments. The proposed inverted repeats (found by Clustal W, Lasergene 17 with default parameters) are marked in blue, below there is the consensus sequence of the repeats.
